# Mapping Levels of Palliative Care Development in 198 Countries: The Situation in 2017

**DOI:** 10.1016/j.jpainsymman.2019.11.009

**Published:** 2020-04

**Authors:** David Clark, Nicole Baur, David Clelland, Eduardo Garralda, Jesús López-Fidalgo, Stephen Connor, Carlos Centeno

**Affiliations:** a School of Interdisciplinary Studies, University of Glasgow, Scotland, United Kingdom; b ATLANTES Global Observatory of Palliative Care, Institute for Culture and Society, University of Navarra, Pamplona, Spain; c Worldwide Hospice Palliative Care Alliance, London, United Kingdom

**Keywords:** Palliative care, hospice, mapping, global development, indicators

## Abstract

**Context:**

Palliative care is gaining ground globally and is endorsed in high-level policy commitments, but service provision, supporting policies, education, and funding are incommensurate with rapidly growing needs.

**Objectives:**

The objective of this study was to describe current levels of global palliative care development and report on changes since 2006.

**Methods:**

An online survey of experts in 198 countries generated 2017 data on 10 indicators of palliative care provision, fitted to six categories of development. Factor analysis and discriminant analysis showed the validity of the categorization. Spearman correlation analyses assessed the relationship with World Bank Income Level (WBIL), Human Development Index (HDI), and Universal Health Coverage (UHC).

**Results:**

Numbers (percentages) of countries in each development category were as follows: 1) no known palliative care activity, 47 (24%); 2) capacity-building, 13 (7%); 3a) isolated provision, 65 (33%); 3b) generalized provision, 22 (11%); 4a) preliminary integration into mainstream provision, 21 (11%); 4b) advanced integration, 30 (15%). Development levels were significantly associated with WBIL (r_S_ = 0.4785), UHC (r_S_ = 0.5558), and HDI (r_S_ = 0.5426) with *P* < 0.001. Net improvement between 2006 and 2017 saw 32 fewer countries in Categories 1/2, 16 more countries in 3a/3b, and 17 more countries in 4a/4b.

**Conclusion:**

Palliative care at the highest level of provision is available for only 14% of the global population and is concentrated in European countries. An 87% global increase in serious health-related suffering amenable to palliative care interventions is predicted by 2060. With an increasing need, palliative care is not reaching the levels required by at least half of the global population.

## Key Message

With an increasing need, palliative care is not reaching the levels required by at least half of the global population. Our analysis of 198 countries casts doubt on the effectiveness of recent global strategies and emphasizes the urgent need for greater palliative care development and implementation, building on the identified infrastructures.

## Background

The delivery of palliative care is seen increasingly as a global health issue. In 2014, the World Health Assembly passed a declaration calling upon all governments to integrate the provision of palliative care into their health plans.^1^ The Lancet Commission Report on Palliative Care and Pain Relief in 2017 estimated that almost half of the people who die each year encounter “serious health-related suffering” that could benefit from palliative care, 80% of them in low- and middle-income countries.^2^ The 2018 Declaration of Astana, focusing on primary care as an aspect of Universal Health Coverage and sustainable development goals, included palliative care across a spectrum of provision that must be accessible to all.^3^

Such high-level policy interventions, framed within the wider discourse of global health, are designed to support the worldwide improvement of palliative care and its integration into health systems. There is growing evidence of the enormous need for palliative care that the world is facing.^4^ The burden of serious health-related suffering will almost double by 2060, with the fastest increases occurring in low-income countries, among older people, and people with dementia. Although it has become common to describe the need for global action to integrate palliative care into health systems as an ethical and economic imperative, palliative care development remains patchy, the field often lacks recognition, there is a dearth of investment, and research evidence to support its global growth is limited.^5^ Much development at the country level continues to be spearheaded by motivated individuals and nongovernmental organizations, often with limited financial, political, and policy influence. Progress is slow and it is unclear whether high-level policy interventions can escalate the speed and volume of palliative care development around the world.^6^

Two WHO studies have thrown some light on palliative care development globally. A 2015 survey^7^ was able to report that 37% of countries had an operational national policy for noncommunicable diseases which included palliative care; palliative care services were financially disadvantaged compared to other noncommunicable disease services; and a large country-income gradient existed for palliative care funding, for oral morphine availability, and the integration of palliative care at the primary levels of the health system. A 2017 survey^8^ reported that 68% of countries had some form of funding for palliative care and approximately one-third of countries responded that palliative care was generally available in both primary health care facilities (35%) and community or home-based care (37%).

We report here on a unique global program of work that has been monitoring country-level development in palliative care, across income categories, for more than a decade, beginning in 2006,^9^ followed up in 2011,^10^ and now updated for 2017. These original studies have contributed significantly to advocacy, planning, and monitoring of palliative care worldwide and complement related work done for smaller numbers of countries by the Economist Intelligence Unit.^11^^,^^12^ In 2014, the second global study formed a key aspect of the evidence base^13^ for the 67th World Health Assembly (WHA) Resolution on Palliative Care, which was supported by all member states. Our goal has been to present an unfolding analysis of global palliative care development over time in ways that can inform policy and advocacy.

The aims of the present study were 1) to allocate each country to one of six categories of palliative care development in 2017 (Table 1) and 2) to track category changes since 2006.

**Table 1:** Six Levels of Palliative Care Development.

Category 1: No known palliative care activity	A country in this category is one where current research reveals no evidence of any palliative care activity.
Category 2: Capacity-building palliative care activity	A country in this category shows evidence of wide-ranging initiatives designed to create the organizational, workforce, and policy capacity for the development of palliative care services, although no service has been established yet. Developmental activities include attendance at, or organization of, key conferences, personnel undertaking external training in palliative care, lobbying of policy makers and Ministries of Health and emerging plans for service development.
Category 3a: Isolated palliative care provision	A country in this category is characterized by the development of palliative care activism that is still patchy in scope and not well supported; sources of funding that are often heavily donor dependent; limited availability of morphine; and a small number of palliative care services that are limited in relation to the size of the population.
Category 3b: Generalized palliative care provision	A country in this category is characterized by the development of palliative care activism in several locations with the growth of local support in those areas; multiple sources of funding; the availability of morphine; several hospice-palliative care services from a range of providers; and the provision of some training and education initiatives by the hospice and palliative care organizations.
Category 4a: Palliative care services at a preliminary stage of integration to mainstream health care services	A country in this category is characterized by the development of a critical mass of palliative care activism in a number of locations; a variety of palliative care providers and types of services; awareness of palliative care on the part of health professionals and local communities; a palliative care strategy that has been implemented and is regularly evaluated; the availability of morphine and some other strong pain-relieving drugs; some impact of palliative care on policy; the provision of a substantial number of training and education initiatives by a range of organizations; and the existence of a national palliative care association.
Category 4b: Palliative care services at an advanced stage of integration to mainstream health care services	A country in this category is characterized by the development of a critical mass of palliative care activism in a wide range of locations; comprehensive provision of all types of palliative care by multiple service providers; broad awareness of palliative care on the part of health professionals, local communities, and society in general; a palliative care strategy that has been implemented and is regularly updated; unrestricted availability of morphine and most strong pain-relieving drugs; substantial impact of palliative care on policy; the existence of palliative care guidelines; the existence of recognized education centers and academic links with universities with evidence of integration of palliative care into relevant curricula; and the existence of a national palliative care association that has achieved significant impact.

## Methods

An open-access protocol^14^ contains a full description of our methods, design, use of indicators, data collection, analysis, and how these have been improved over previous iterations of the study, taking into account of published commentary about limitations of the method.^15^^,^^16^

### Data Sources

We designed and piloted an online questionnaire to be completed by in-country experts. These were defined as follows:

1. Representatives of the national in-country hospice-palliative care association or nearest professional association (e.g., society for palliative medicine, hospice forum). The person should have an established administrative and/or leadership role in the organization making them a reliable source of information.
2. Academic experts with known interests and research experience in hospice-palliative care development in-country and/or beyond as evidenced by peer-reviewed publications. The person should have an established academic role in hospice-palliative care research or education making them a reliable source of information.
3. Policy specialists (in or outside government) with experience of and/or responsibility for hospice-palliative care delivery in-country. The person should have an established policy role relating to hospice-palliative care making them a reliable source of information.

We also used two sets of additional data from third-party sources: country populations^17^ and country-level opioid consumption.^18^ Our study population included 198 territories, comprising the 193 Member States of the United Nations (UN), two observer states, along with Kosovo, Somaliland, and Taiwan, China. We surveyed a total of 560 experts from 179 (90%) countries for which contacts could be found.

Respondents were identified by global and regional palliative care associations, from named persons in published regional atlases of palliative care, and from the wider literature. For countries where no questionnaire data were obtained or where the questionnaire data were incomplete, we supplemented where possible by systematic review of the available published literature or extraction of data from Regional Palliative Care Atlases published since 2011.^19^, ^20^, ^21^, ^22^

### Analysis

**Fig. 1:**
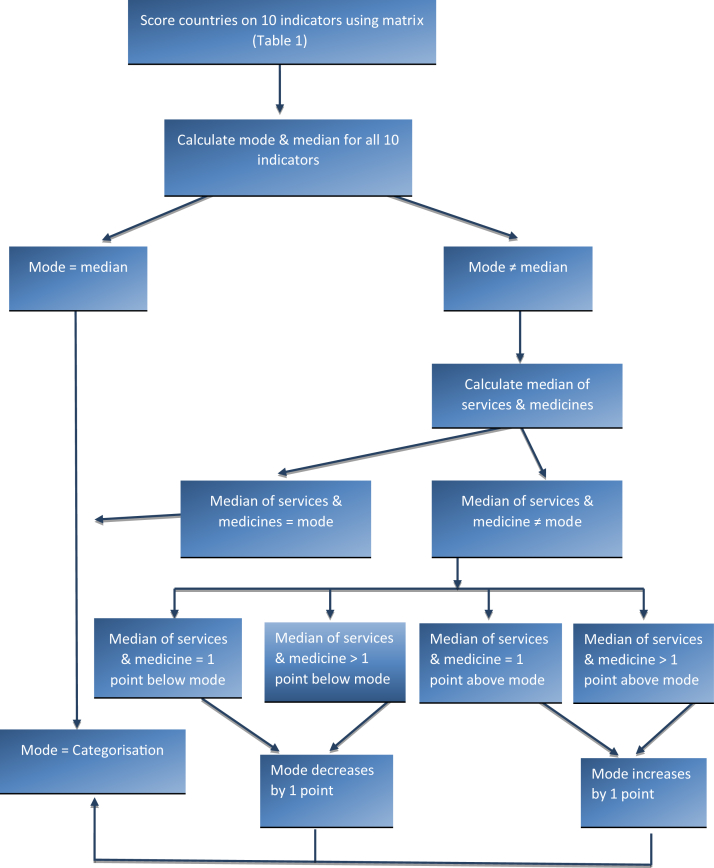
Scoring algorithm to determine categories of palliative care development.

We established 10 indicators of palliative care development, drawn from the emerging literature^23^ and linked to specific questionnaire items (Table 2). For each indicator, we established an outcome range of six levels, fitted to the palliative care development categories and the definitions adopted. This enabled each country where complete data existed to be assigned a numerical value between zero (Category 1) and five (Category 4b) across each of the 10 indicators. Where data had to be supplemented from documentary sources, we allocated scores to the missing indicators based on research team members' assessment of the sources. The scores were then applied to an analytic algorithm (Fig. 1), using the mode and the median of these indicators. Where mode and median were coincident, this determined the country's overall level of categorization. Where they were not, the mode was moderated either upward or downward by one category based on whether the median value of four specific selected indicators, judged to be the most “consequential” for palliative care development, was higher or lower than the mode. These four consequential indicators were as follows: the availability and consumption of opioids, and the number and geographic distribution of services.

**Table 2:** Indicators of Palliative Development

WHO dimension	Score	Categories					
		0	1	2	3	4	5
	Indicator	Category 1 No known PC activity	Category 2 Capacity-building PC activity	Category 3a Isolated PC provision	Category 3b Generalized PC provision	Category 4a PC services at preliminary stage of integration	Category 4b PC services at advanced stage of integration
Services (Q15)	Provision of services^a^	No evidence/don't know	No evidence	0–0.49 per 100,000	0.5–0.99 per 100,000	1.0–1.49 per 100,000	1.5 and more per 100,000
(Q17)	Geographical spread of services	No evidence/don't know	In progress	1–4	5–6	7–8	9–10
Funding (Q18)	Range of available funding sources for palliative care	No evidence/don't know	Direct payments	Direct payments, donor	Donor, institutions & partial NHS (pilot projects)	NHS participates in the funding on a regular basis	Mainly by NHS or health finance system
Strategy or national plan (Q19 a/e/f/g/k)	Existence of national strategy or plan for palliative care	No evidence/don't know	No reference	Reference to PC in national strategies for cancer, AIDS, and/or other noncommunicable diseases	Strategy or national plan specific to PC	PC strategy implemented and evaluated	PC strategy implemented and updated OR Desk at Ministry of Health
Law (Q19 b/c/d)	Existence of legal provision to support palliative care	No evidence/don't know	No reference	Establishment in progress of any reference (decrees/norms) but not national law—could be regional law (e.g., Germany)	Any reference (decrees/norms) but not national law—could be regional law (e.g., Germany)	References to PC in national laws	Standalone PC law or recognition of PC as a right in top law or the constitution of the country
Medicine (Q21/22)	Availability of morphine and other strong opioids	No evidence/don't know	Not available	Morphine occasionally available	Morphine usually available	Morphine always available, other opioids usually available	Any kind of strong opioids always available
	Country consumption of morphine per capita (2015)	No evidence/don't know	0.0001–0.2399 (Quartile 1)	0.2400–1.0387 (Quartile 2)	1.0388–3.9857 (Quartile 3)	>3.9857 (Quartile 4)	>3.9857 (Quartile 4) and any kind of strong opioids always available
Education (Q23)	Training programs for professionals in palliative care	No evidence/don't know	Professionals receive training abroad, basic courses are available in the country	Informal process of training for palliative care professionals available in the country	Establishment of official process of palliative medicine specialization in the country in progress	Official process of palliative medicine specialization available in the country	Substantial number of professionals certified
(Q24/25)	Education for prequalification doctors/nurses	No evidence/don't know	Teaching by nonprofit sector and/or hospice organizations	Teaching is available at hospitals/medical centers/university hospitals or through Ministry of Health	Teaching is available in the primary care sector	Universities provide PC training	Universities provide PC training and palliative medicine is a recognized medical specialty or subspecialty
Vitality (Q19 hours/i/j/l/m/n/o)	Existence of meetings, associations, journals, conferences, guidelines, collaborations in palliative care	No evidence/don't know	Evidence of PC professional or political meetings	Existence of a national PC association or establishment in progress	Existence of at least one of the following: a national journal, palliative care directory, standards or guidelines and national conference AND a national PC association	Existence of at least two of the following: a national journal, palliative care directory, standards or guidelines and national conference AND a national PC association	Existence of at least two of the following: a national journal, palliative care directory, standards or guidelines and national conference AND a national PC association as well as evidence of professional co-operation with other specialties outside PC (national or international)

a This indicator relates to the total number of palliative care services operating in a country. These include, but are not limited to, freestanding hospices with or without inpatient beds, hospices that are a part of public or NGO hospitals, home care teams, palliative care support teams in hospitals, palliative care inpatient and outpatient facilities, pediatric palliative care hospices and services. The focus is on services that are providing specialized/specialist palliative care as their primary mission. A palliative care service provider organization may have more than one local service in operation, so the number of palliative care services in a country may be greater than the number of provider organizations. (This definition was included in the questionnaire).

By this process, each country was allocated to one of the six predetermined categories of palliative care development. Factor analysis and discriminant analysis were performed to justify the categorization validity.

Spearman correlation coefficients were computed to discover significant associations between level of palliative care development and World Bank Income Level (WBLI), Human Development Index (HDI), and Universal Health Coverage (UHC).

For purposes of comparison over time, we combined Categories 3a and 3b, 4a and 4b, from the 2011 and 2017 studies, controlled for new states recognized by the UN since the first world map of palliative care was developed, and excluded jurisdictions (mainly UN-associated territories) not included in the present study.

### Findings

**Fig. 2:**
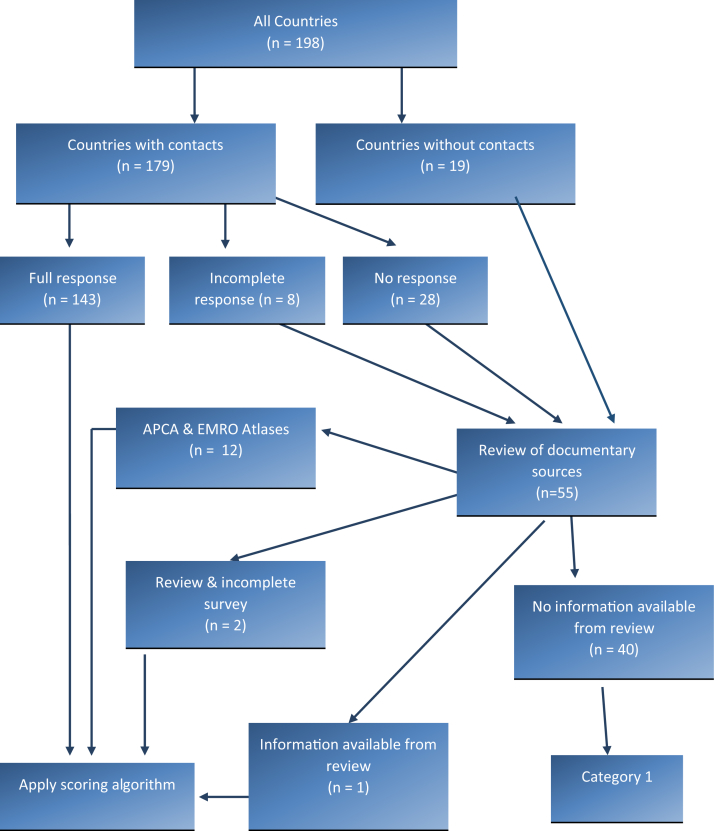
Data sourcing process.

Completed questionnaires were obtained from 143 (76%) countries; for eight countries (4%), the questionnaire data were incomplete; 28 (14%) countries contacted did not reply and provided no questionnaire data; for 19 (10%) countries, we were unable to identify a contact. Population data were obtained for 198 countries, but opioid consumption data were not available for 45 countries. For the 55 countries with missing or incomplete questionnaire data, we substituted, where possible, with information from documentary sources, and categorized the 198 countries as shown in Fig. 2. Countries where no data were available were placed in Category 1.

### Categorization of Level of Palliative Care Development at 2017

**Fig. 3:**
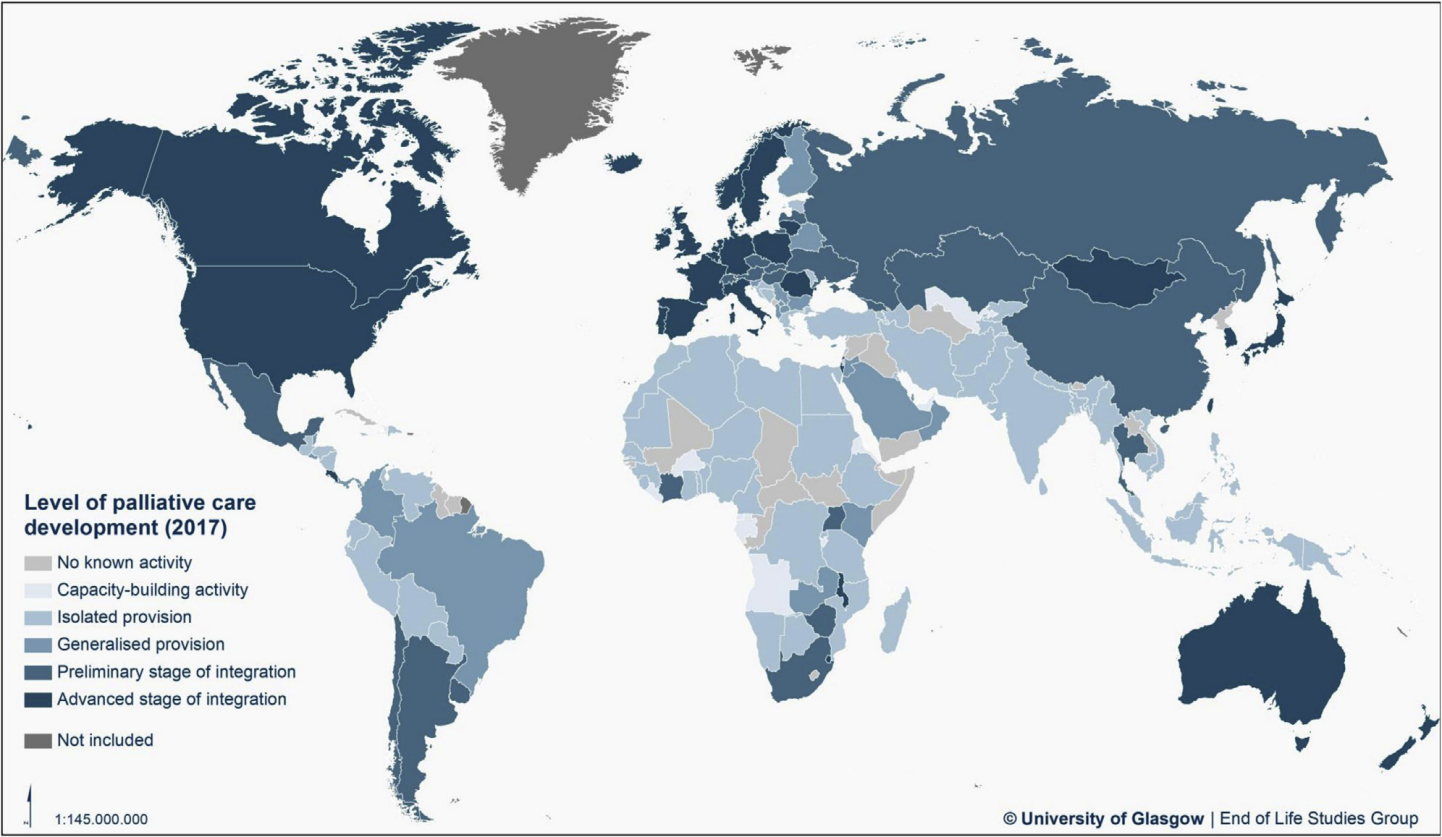
Global levels of palliative care development.

Table 3 and the map at Fig. 3 show the levels of palliative care development for 198 countries. Only 30 countries (15%) in the world are in the highest level of palliative care development. These countries represent 14% of the world population. A further 21 countries (11%) have high levels of palliative care development, but not across all indicators. They comprise 28% of the world population.

**Table 3:** Level of Palliative Care Development in 2017 by Country, Population, WHO Region, and World Bank Income Level

Category *Number of Countries (%): Total Population (% of World Population)*	WHO Region	Countries
Category 1: No known palliative care activity *47 countries (24%);* *235 million people (3.1% of world population)*	Africa	Cape Verde, Central African Republic, Chad, Comoros, Congo (Republic), Guinea-Bissau^a^, Lesotho, Mali, Seychelles, South Sudan
	Americas	Antigua & Barbuda, Cuba, Dominica^a^, Grenada^a^, Guyana, Saint Lucia, St Kitts & Nevis^a^, St Vincent & the Grenadines^a^, Suriname^a^
	Eastern Mediterranean	Djibouti, Iraq, Somalia, Somaliland, Syria^a^, Yemen
	Europe	Andorra, Kosovo^a^, Monaco, Montenegro, San Marino^a^, Turkmenistan, Vatican City
	South-East Asia	Bhutan, Maldives^a^, North Korea, Timor l'Este
	Western Pacific	Brunei, Kiribati, Laos, Marshall Islands, Micronesia^a^, Nauru^a^, Palau^a^, Solomon Islands^a^, Tonga, Tuvalu^a^, Vanuatu^a^
Category 2: Capacity-building palliative care activity *13 countries (7%);* *126 million people (1.7% of world population)*	Africa	Angola, Burkina Faso, Burundi, Equatorial Guinea, Eritrea, Gabon, Liberia, Sao Tome e Principe
	Americas	Bahamas, Haiti
	Eastern Mediterranean	United Arab Emirates
	Europe	Uzbekistan
	South-East Asia	—
	Western Pacific	Samoa
Category 3a: Isolated palliative care provision *65 countries (33%);* *3597 million people (47.7% of world population)*	Africa	Algeria, Benin, Botswana, Cameroon, Congo (DR), Ethiopia, Ghana, Guinea, Madagascar, Mauretania, Mauritius, Mozambique, Namibia, Niger, Nigeria, Rwanda, Senegal, Sierra Leone, Tanzania, Togo
	Americas	Bolivia, Dominican Republic, Ecuador, Guatemala, Honduras, Jamaica, Nicaragua, Paraguay, Peru, Trinidad & Tobago, Venezuela
	Eastern Mediterranean	Afghanistan, Bahrain, Egypt, Iran, Kuwait, Lebanon, Libya, Morocco, Pakistan, Palestine, Sudan, Tunisia
	Europe	Armenia, Azerbaijan, Bosnia & Herzegovina, Croatia, Estonia, Greece, Kyrgyzstan, Moldova, Tajikistan, Turkey
	South-East Asia	Bangladesh, India, Indonesia, Myanmar, Nepal, Sri Lanka
	Western Pacific	Cambodia, Fiji, Malaysia, Papua New Guinea, Philippines, Vietnam
Category 3b: Generalized palliative care provision *22 countries (11%);* *426 million people (5.7% of world population)*	Africa	Gambia, Kenya, Zambia
	Americas	Belize, Brazil, Colombia, El Salvador, Panama
	Eastern Mediterranean	Jordan, Oman, Qatar, Saudi Arabia
	Europe	Albania, Belarus, Bulgaria, Cyprus, Finland, Luxembourg, Macedonia, Malta, Serbia, Slovenia
	South-East Asia	—
	Western Pacific	—
Category 4a: Palliative care at preliminary stage of integration *21 countries (11%);* *2083 million people (27.6% of world population)*	Africa	Côte d’Ivoire, South Africa, Uganda, Zimbabwe
	America	Argentina, Chile, Mexico, Uruguay
	Eastern Mediterranean	—
	Europe	Austria, Czech Republic, Georgia, Hungary, Kazakhstan, Latvia, Russia, Slovakia, Switzerland, Ukraine
	South-East Asia	Thailand
	Western Pacific	China, Singapore
Category 4b: Palliative care at advanced stage of integration *30 countries (15%);* *1074 million people (14.2% of world population)*	Africa	Malawi, Swaziland
	America	Barbados, Canada, Costa Rica, United States of America
	Eastern Mediterranean	—
	Europe	Belgium, Denmark, France, Germany, Iceland, Ireland, Israel, Italy, Liechtenstein, Lithuania, Mongolia, The Netherlands, Norway, Poland, Portugal, Romania, Spain, Sweden, United Kingdom
	South-East Asia	—
	Western Pacific	Australia, Japan, New Zealand, South Korea, Taiwan

a Denotes countries placed in Category 1 because no contacts for survey were identified.

Close to one-quarter of countries (47, 24% of the total) have no known palliative care activity. These countries represent 3.1% of the global population.

Between these extremes, there are 13 countries (7%) that are still building capacity in advance of actual palliative care service provision and 87 countries (44%) where development and delivery are localized to a significant degree and lack sufficient integration with the wider health and social care system to achieve high coverage. These 87 countries of limited palliative care development represent 53% of the global population.

Table 4 shows the relationship between the level of palliative care development and the United Nations Human Development Index,^24^ World Bank Income level,^25^ Universal Health Coverage,^26^ and WHO region.^27^ There is a strong tendency for countries that are more developed on these measures to have higher levels of palliative care development, with 64% of countries at the highest levels of human development falling in Categories 4a and 4b. There are however outliers at all levels of development, with a small number of highly developed countries having little palliative care provision, and four countries that are at the lowest level of human development but are nevertheless placed in the highest two categories of palliative care provision.

**Table 4:** Level of Palliative Care Development by Country-Level Indicators

Indicator	Category 1		Category 2		Category 3a		Category 3b		Category 4a		Category 4b		Total
	No.	%	No.	%	No.	%	No.	%	No.	%	No.	%	No.
Human Development Index level													
Very high	3	5	2	3	6	10	10	17	12	21	25	43	58
High	14	26	3	6	21	40	8	15	5	9	2	4	53
Medium	10	26	3	8	21	54	3	8	1	3	1	3	39
Low	11	29	5	13	17	45	1	3	3	8	1	3	38
No Human Development Index	9	90	—	—	—	—	—	—	—	—	1	10	10
World Bank Income level													
High	8	13	2	3	6	10	9	15	10	17	25	42	60
Upper-middle	16	28	3	5	21	37	9	16	6	11	2	4	55
Lower-middle	11	25	3	7	22	50	3	6	3	7	2	5	47
Low	10	29	5	15	15	44	1	3	2	6	1	3	33
No World Bank Income level	2	67	—	—	1	33	—	—	—	—	—	—	3
Universal Health Care Index quintile													
Q5 (high)	2	6	0	0	2	6	7	19	4	11	21	58	36
Q4	2	6	2	6	12	34	7	20	9	26	3	9	35
Q3	10	26	1	3	17	44	4	10	5	13	2	5	39
Q2	11	31	4	11	14	40	4	11	1	3	1	3	35
Q1 (low)	10	26	6	16	19	50	—	—	2	5	1	3	38
No Universal Health Care Index	12	80	—	—	1	7	—	—	—	—	2	13	15
WHO region													
Africa	10	21	8	17	20	43	3	6	4	9	2	4	47
America	9	26	2	6	11	31	5	14	4	11	4	11	35
Eastern Mediterranean	6	26	1	4	12	52	4	17	—	—	—	—	23
Europe	7	13	1	2	10	18	10	18	10	18	18	32	56
South-East Asia	4	36	—	—	6	55	—	—	1	9	—	—	11
Western Pacific	11	42	1	4	6	23	—	—	2	8	6	23	26
Total	47	24	13	7	65	33	22	11	21	11	30	15	198

Percentages are of row totals.

We compared for the first time the level of palliative care development with an index of progress toward UHC, as defined by the UN Sustainable Development Goal Target 3.8—that all people receive the essential health services they need, without being exposed to financial hardship. Table 4 shows a close relationship between the level of development of palliative care services and the level of UHC. Over half (58%) of the 36 countries in the top quintile for UHC service coverage are at the highest level of palliative care development. Similarly, only four countries in the highest categories of palliative care development are in the lowest two UHC quintiles or countries with no UHC Index.

**Supplementary Figure 1:**
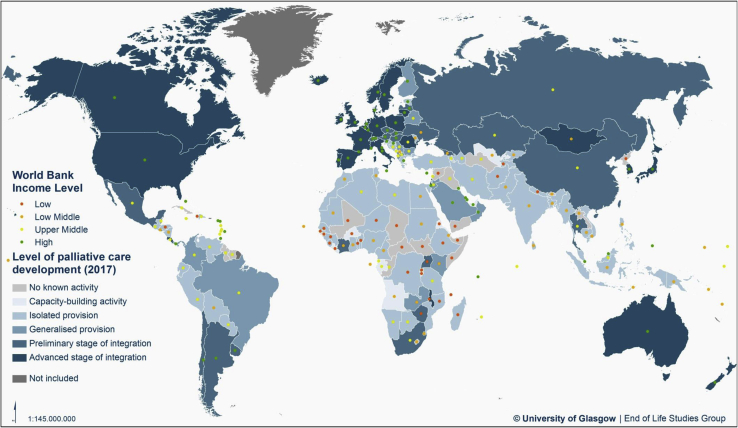
Levels of palliative care development and World Bank income levels.

**Supplementary Figure 2:**
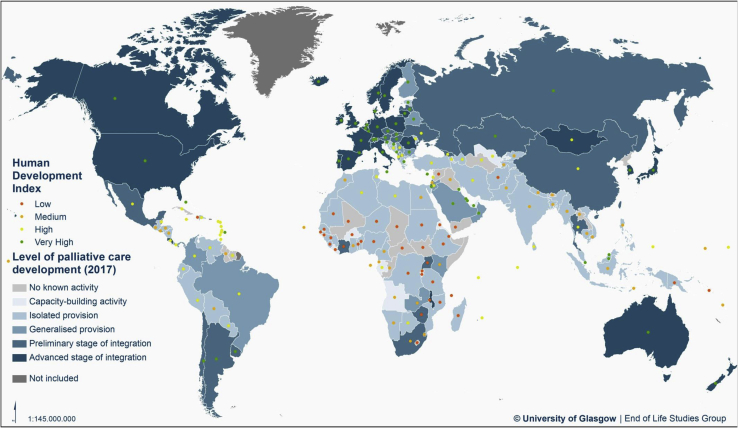
Levels of palliative care development and UN Human Development Index Levels.

Supplementary Figure 1, Supplementary Figure 2, and 3 contain maps that reveal for each country the level of palliative care development and the WBIL, SDI, and UHC Index, respectively.

Spearman correlation analyses of palliative care development category against these indicators showed level of development to be significantly associated with WBIL (r_S_ = 0.4785), UHC (r_S_ = 0.5558), and HDI (r_S_ = 0.5426) with *P* < 0.001, with a moderate size effect in each case.

Among the WHO geo-health regions, Europe has 32 of 56 countries in the highest level of development, whereas the Eastern Mediterranean and South-East Asia regions have no countries in this category.

### Change Over Time, 2006–2017

We assessed country-level changes in palliative care development over a period of 11 years (Table 5).

**Table 5:** Levels of Palliative Care Development for 198 Countries and Extent of Net Change: 2006, 2011, 2017 (Four-Part Typology)

Country Category	World Map 1 (2006)	Change WM1→2	World Map 2 (2011)	Change WM2→3	World Map 3 (2017)	Total Change WM1→3
Number of countries						
Category 1	51	−2	49	−2	47	−4
Category 2	38	−17	21	−8	13	−25
Category 3	71	11	82	5	87	16
Category 4	34	8	42	9	51	17
Total	194	0	194	4	198	4
% of countries						
Category 1	26.3	−1.0	25.3	−1.6	23.7	−2.6
Category 2	19.6	−8.8	10.8	−4.2	6.6	−13.0
Category 3	36.6	5.7	42.3	1.6	43.9	7.3
Category 4	17.5	4.1	21.6	4.2	25.8	8.3
Total	100	—	100	—	100	—
% of world population						
Category 1	4.2	0.1	4.3	−1.2	3.1	−1.1
Category 2	8.2	−5.3	2.9	−1.2	1.7	−6.5
Category 3	69.8	−14.7	55.1	−1.8	53.3	−16.5
Category 4	17.2	19.9	37.1	4.7	41.8	24.6
Other territories	0.6	0.0	0.6	−0.5	0.1	−0.5
Total	100.0	—	100.0	—	100.0	—

Countries included in the present study were limited to the 193 UN Member States, two observer states, plus Taiwan, Kosovo, and Somaliland. Earlier surveys did not include Taiwan, Kosovo, and Somaliland, or South Sudan which became a UN Member in 2011.

The number of countries at the highest level of palliative care development (combined Category 4) has increased by 17 since 2006, representing an additional 24.6% of the world population. As the number of countries in combined Category 3 has also increased (by 16 since 2006), the number of people living in countries at this level has fallen, as larger countries have moved from Category 3 to Category 4.

The number of countries at the lowest level of palliative care development (Category 1) has only declined slightly, with four fewer countries in this category since 2006. Just 3.1% of the world population live in Category 1 countries.

**Fig. 4:**
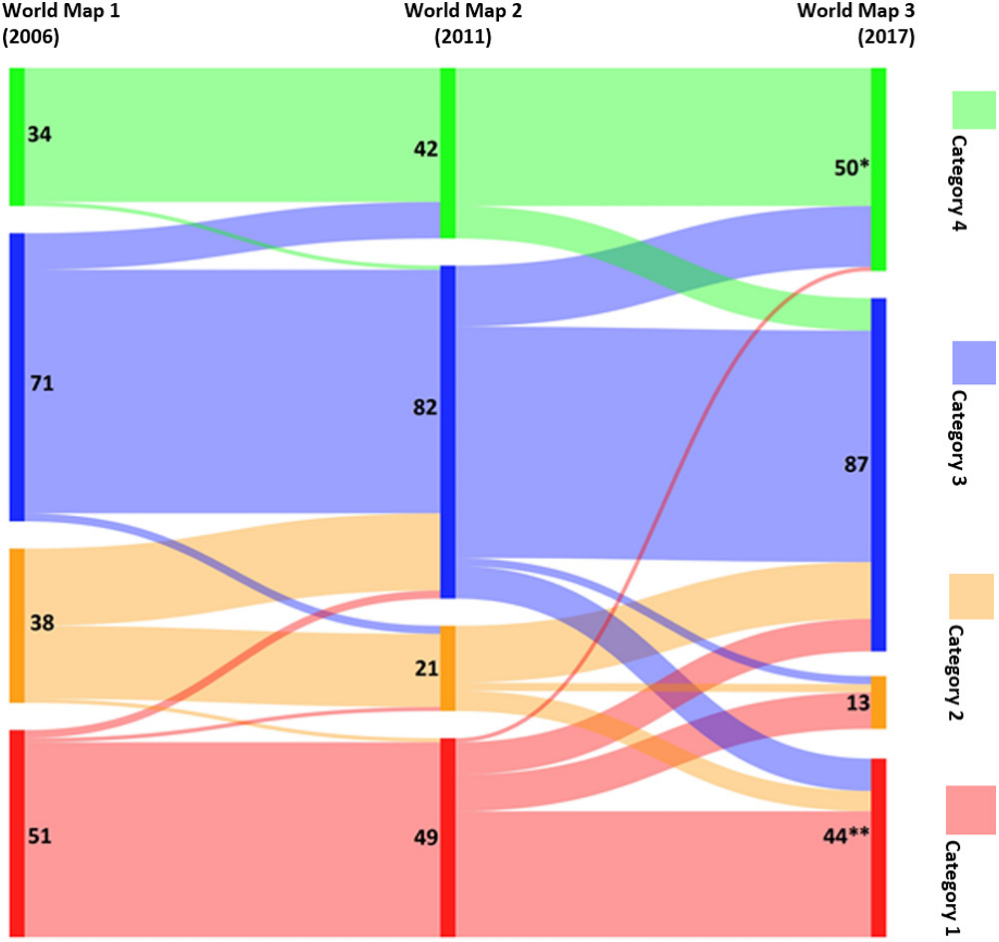
Movement of countries between palliative care development levels (four-part typology). *One additional country in Category 4 was not included in WM1 or 2. **Three additional countries in Category 1 were not included in WM1 or 2.

In Fig. 4, we show the volume and direction of movement between the four aggregated categories of palliative care development from one survey to the next. In Supplementary Table 1, we provide the underlying data, showing the categorization of each country across the three iterations of the study.

Between the first, second, and third surveys, there was net upward movement of countries across the four categories. Nine countries that had been in Category 3 in 2006 moved to Category 4 in 2011; 15 countries made this transition between 2011 and 2017. There has also been some downward movement, in particular between 2011 and 2017, with eight countries moving down from Category 4, 10 from Category 3, and five from Category 2 in this period.

A number of countries appear to have experienced substantial shifts in their level of palliative care development, moving by more than one category. Eight countries in Category 1 in 2011 were in Category 3 in 2017; another eight countries had also made this transition in the opposite direction. One country that had been placed in the lowest category in 2011 was allocated to the highest category in 2017—a small high-income country for which no evidence on the level of palliative care development had previously been identified.

The number of countries in the world with some form of palliative care service has risen from 105 (2006) to 124 (2011) to 138 (2017). The number of countries with some level of integration of palliative care into mainstream provision increased from 34 (2006), to 42 (2011) to 51 (2017).

### Statistical Analysis of the Categorization

Factor analysis is a statistical technique to discover different sets of variables explaining the same feature. Each set of variables (in this case, indicators of palliative care development) is summarized in a new variable called a factor. Frequently after an ordinary factor analysis, the groups are not clearly determined and a second step is needed to identify them. This second step is based on rotations of the factors, considered as vectors in the space. There are a number of methods for rotating the factors. In our case, the so-called “varimax rotation” gave the best description of two sets of indicators.

**Fig. 5:**
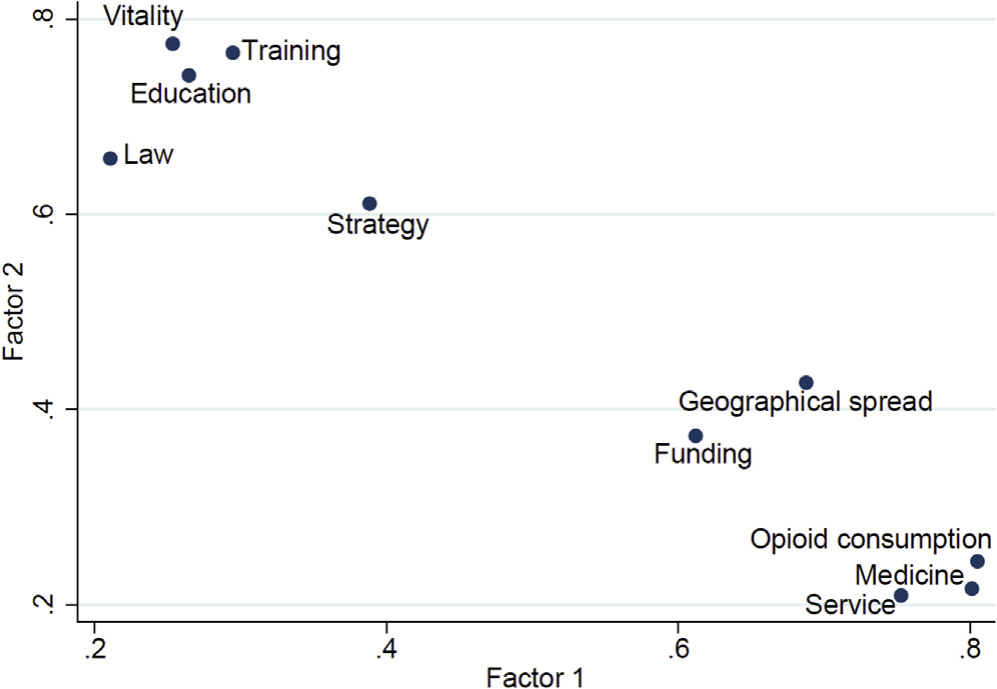
Loadings of the factor analysis with varimax orthogonal rotation. Points relate to indicators listed in Table 2.

Factor analysis was performed for application of the 10 indicators, to check their robustness (Fig. 5). The test included 140 countries with complete data for all 10 indicators (including opioid consumption). One factor explained more than 50% of the variability and the load was around 0·7, giving strength to the categorizations.

After varimax rotation, opioid consumption and available medicines showed up most strongly in the first factor from the four “consequential” indicators (along with “funding” also); the remaining five indicators appeared in the second factor.

After this, we considered the two obtained factors to check whether the groups identified were coherent with the indicators or not. To do that, we used a machine-supervised learning technique based on Fisher's linear discriminant analysis. Priors proportional to the group sizes were used because this option does not need the assumption of normality in each group. This confirmed the appropriateness of the indicators for making the categorization of palliative care development, with the analysis verifying the classification in 83% of the 140 countries (Table 6).

**Table 6:** Fisher's Linear Discriminant Analysis for 140 Countries

True Categories Classified by the Algorithm	Map Categories Classified by the Factorial Analysis					
	1	2	3a	3b	4a	4b
1						
3	**2**	1	0	0	0	0
100%	**66.67%**	33.33%	0%	0%	0%	0%
2						
8	1	**4**	3	0	0	0
100%	12.50	**50.00**	37.50	0%	0%	0%
3a						
59	0	0	**58**	1	0	0
100%	0%	0%	**98.31**	1.69	0%	0%
3b						
21	0	0	3	**14**	3	1
100%	0%	0%	14.29	**66.67**	14.29	4.76
4a						
26	0	0	1	6	**17**	2
100%	0%	0%	3.85	23.08	**65.38**	7.69
4b						
23	0	0	0	0	2	**21**
100%	0%	0%	0%	0%	8.70%	**91.30%**
Total						
140	3	5	65	21	22	24
100%	2.14%	3.57%	46.43%	15.00%	15.71%	17.14%
Priors	0.0214	0.0571	0.4214	0.1500	0.1857	0.1643

140 countries with complete data for all 10 indicators (complete questionnaire plus opioid consumption) were included in this supervised classification. Of the total 140 countries, the classification of 116 countries (83%) was verified by the discriminant analysis.

Entries in bold indicate the numbers and proportions of correctly classified countries in each category.

### Limitations

We are in no doubt that our approach continues to have significant limitations, despite the improvements we have made to the method of data collection and to the analysis. We describe these limitations and the improvements in detail in the study protocol.^14^

There is a debate in the literature^7^^,^^14^ about the merits of using palliative care specialists or government sources to obtain the kind of data we report here. Both have their limitations. The former, although close to the field, often over long periods, may underrepresent or overrepresent the available palliative care provision for perceived strategic reasons; the latter are often less close to the field and can change roles rapidly, leading to a lack of cumulative knowledge. In the present study, we used both sources but privileged the former source where there were two options.

Data limitations were compounded by language constraints. Questionnaires were only available in three European languages.

Missing data were a problem we sought to overcome collectively as a research team. On a case-by-case basis, we searched our extensive records of palliative care development publications, published regional atlases of palliative care, gray literature, and Internet sources, to make informed and moderated judgments that would allow the scoring of indicators to be completed. We acknowledge the potential biases of this but argue that the benefits of a fuller and more detailed picture outweigh the limitations.

It has been observed that although our approach gives an overall level of palliative care development for a country, in many instances, levels of development can vary significantly within country, by region or locality. We sought to address this by including an indicator of geographic coverage, but we acknowledge that more work can be done to establish regional variations in palliative care development, especially in large and populous countries, or where responsibility for palliative care may be devolved to subnational governments.

We fully acknowledge that rigorously tested indicators of palliative care access and development do not yet exist, that collaborative efforts to develop such reliable indicators and reach consensus upon them should continue,^28^ and that the project we report here remains (as we have stated previously) a work in progress and one which we seek continually to improve.

## Discussion

Palliative care “resolutions” continue to appear from global health organizations, policy makers, and activists,^29^ but progress toward universal palliative care coverage is hugely constrained. Ours is the only global palliative care development study of its kind. We show that the world population is effectively split down in the middle: between those who live in countries with reasonably robust systems of specialized palliative care delivery, and those who do not. The countries with the highest levels of palliative care development contain 41.8% of the world population and are concentrated in the Global North, though not exclusively, while 80% of the need for palliative care is in low- and middle-income countries. 53.3% of the world's population is in countries with very limited palliative care development mainly in the Global South, though not exclusively. The remainder of the global population (4.8%) is located in countries that have no known palliative care activity or are only at the level of capacity building, and in territories that were not included in the survey (0.1%).

The Lancet Commission on Palliative Care and Pain Relief highlighted an “access abyss” that separates those in need of palliative care from available services. Our study reveals the fragile and moderate palliative care assets and service infrastructure on which the goals of the Commission will be reliant as it seeks to build greater palliative care capacity within mainstream provision. It is likely that diffusion of the essential package of services called for in the Lancet report will be very difficult to deliver without increased investment in specialized palliative care infrastructure, as a platform from which wider implementation can occur.

Our comments on change over time are offered with a sense of caution. Improvements made in methods of data collection and analysis for each iteration of the study do inhibit comparisons over the three time periods on which we report. Nevertheless, in presenting these data, we are able to provide a unique insight into the slowly evolving development of palliative care provision globally. At the same time, it is important to acknowledge that assignment to a high level of palliative care development leaves no room for complacency, nor should assignment to a low level lead to resignation and resentment. Indeed, we know, from the wide engagement with the results of our earlier mapping studies, that there is a significant collective will in the global palliative care field to see improvement for all countries and to share knowledge and experience to that end. Similarly, although we remain focused on measuring and mapping the development of specialized palliative care services, there is growing interest in monitoring the development of palliative care delivery within mainstream health and social care provision, across primary and tertiary settings, and in the context of numerous medical specialties. These two approaches are complementary to one another.

For the first time, we have used the measure of percentage of the global population, rather than number of countries alone, when assessing coverage of the specific levels of palliative care development. Although the two lowest categories contain 60 countries—almost a third of the total—these account for less than 5% of the global population. At the same time, 87 countries are in categories with operational palliative care, but with weak development, and these make up more than a half of the world population.

With a few exceptions, views about optimal palliative care provision originate in the Global North, where they are frequently seen as a “gold standard” which can somehow be “rolled out” to the Global South, subject only to appropriate resources being made available for implementation.^30^^,^^31^ The veracity of this assumption has to be brought into question by the evidence shown here of slow progress in palliative care development in the poorer countries of the world. Although we continue to applaud efforts to ensure that no one should be left behind in the development of robust palliative care systems, our data might also provoke a debate on whether current global health strategies for palliative care are working. Meanwhile, just 30 countries, comprising less than 15% of the global population, have access to the very highest level of palliative care provision.

## Figures and Tables

**Supplementary Table 1:** Country Palliative Care Development Categories Over Three Global Surveys

Countries	2006^a^	2011	2017
Afghanistan	1	1	3a
Albania	3	3b	3b
Algeria	2	2	3a
Andorra	1	1	1
Angola	1	3a	2
Antigua & Barbuda	1	1	1
Argentina	4	3b	4a
Armenia	3	3a	3a
Australia	4	4b	4b
Austria	4	4b	4a
Azerbaijan	3	2	3a
Bahamas	2	2	2
Bahrain	2	3a	3a
Bangladesh	3	3a	3a
Barbados	3	3a	4b
Belarus	3	3b	3b
Belgium	4	4b	4b
Belize	2	3a	3b
Benin	1	1	3a
Bhutan	1	1	1
Bolivia	2	2	3a
Bosnia & Herzegovina	3	3b	3a
Botswana	3	3a	3a
Brazil	3	3a	3b
Brunei	2	3a	1
Bulgaria	3	3a	3b
Burkina Faso	1	1	2
Burundi	1	1	2
Cambodia	2	3a	3a
Cameroon	3	3a	3a
Canada	4	4b	4b
Cape Verde	1	1	1
Central African Republic	1	1	1
Chad	1	1	1
Chile	4	4a	4a
China	3	4a	4a
Colombia	3	3a	3b
Comoros	1	1	1
Congo (DR)	2	2	3a
Congo (Republic)	3	3a	1
Costa Rica	4	4a	4b
Côte d’Ivoire	2	3b	4a
Croatia	3	3b	3a
Cuba	3	3a	1
Cyprus	3	3b	3b
Czech Republic	3	3b	4a
Denmark	4	4a	4b
Djibouti	1	1	1
Dominica	2	2	1
Dominican Republic	3	3a	3a
Ecuador	3	3a	3a
Egypt	3	3a	3a
El Salvador	3	3a	3b
Equatorial Guinea	1	1	2
Eritrea	1	1	2
Estonia	3	3a	3a
Ethiopia	2	3a	3a
Fiji	2	2	3a
Finland	4	4a	3b
France	4	4b	4b
Gabon	1	1	2
Gambia	3	3a	3b
Georgia	3	3b	4a
Germany	4	4b	4b
Ghana	2	3a	3a
Greece	3	3a	3a
Grenada	1	1	1
Guatemala	3	3a	3a
Guinea	1	1	3a
Guinea-Bissau	1	1	1
Guyana	3	3a	1
Haiti	2	2	2
Honduras	3	2	3a
Hungary	4	4a	4a
Iceland	4	4b	4b
India	3	3b	3a
Indonesia	3	3a	3a
Iran	2	3a	3a
Iraq	3	3a	1
Ireland	4	4b	4b
Israel	4	4a	4b
Italy	4	4b	4b
Jamaica	3	3a	3a
Japan	4	4b	4b
Jordan	3	3b	3b
Kazakhstan	3	3a	4a
Kenya	4	4a	3b
Kiribati	1	1	1
Kosovo^b^	N/A	N/A	1
Kuwait	2	3a	3a
Kyrgyzstan	3	3a	3a
Laos	1	1	1
Latvia	3	3a	4a
Lebanon	2	3a	3a
Lesotho	2	3a	1
Liberia	1	1	2
Libya	1	1	3a
Liechtenstein	1	1	4b
Lithuania	3	3b	4b
Luxembourg	3	4a	3b
Macedonia	3	3a	3b
Madagascar	2	2	3a
Malawi	3	4a	4b
Malaysia	4	4a	3a
Maldives	1	1	1
Mali	1	3a	1
Malta	3	3b	3b
Marshall Islands	1	1	1
Mauritania	1	1	3a
Mauritius	2	2	3a
Mexico	3	3a	4a
Micronesia	1	1	1
Moldova	3	3a	3a
Monaco	1	1	1
Mongolia	4	4a	4b
Montenegro	1	2	1
Morocco	3	3a	3a
Mozambique	2	3a	3a
Myanmar	3	3a	3a
Namibia	2	3a	3a
Nauru	1	1	1
Nepal	3	3b	3a
The Netherlands	4	4a	4b
New Zealand	4	4a	4b
Nicaragua	2	2	3a
Niger	1	1	3a
Nigeria	3	3a	3a
North Korea	1	1	1
Norway	4	4b	4b
Oman	2	2	3b
Pakistan	3	3a	3a
Palau	1	1	1
Palestine	2	2	3a
Panama	3	3a	3b
Papua New Guinea	2	2	3a
Paraguay	2	3a	3a
Peru	3	3a	3a
Philippines	3	3a	3a
Poland	4	4b	4b
Portugal	3	3b	4b
Qatar	2	2	3b
Romania	4	4b	4b
Russia	3	3a	4a
Rwanda	2	3a	3a
Saint Lucia	2	3a	1
Samoa	1	1	2
San Marino	1	1	1
Sao Tome e Principe	1	1	2
Saudi Arabia	3	3a	3b
Senegal	1	1	3a
Serbia	3	4a	3b
Seychelles	2	2	1
Sierra Leone	3	3a	3a
Singapore	4	4b	4a
Slovakia	3	4a	4a
Slovenia	4	4a	3b
Solomon Islands	1	1	1
Somalia	1	1	1
Somaliland^b^	N/A	N/A	1
South Africa	4	4a	4a
South Korea	3	3a	4b
South Sudan^b^	N/A	N/A	1
Spain	4	4a	4b
Sri Lanka	3	3a	3a
St Kitts & Nevis	1	1	1
St Vincent & the Grenadines	1	1	1
Sudan	2	3a	3a
Suriname	2	2	1
Swaziland	3	3b	4b
Sweden	4	4b	4b
Switzerland	4	4b	4a
Syria	1	1	1
Taiwan^b^	N/A	N/A	4b
Tajikistan	2	2	3a
Tanzania	3	4a	3a
Thailand	3	3a	4a
Timor l'Este	1	1	1
Togo	1	1	3a
Tonga	1	1	1
Trinidad & Tobago	3	3a	3a
Tunisia	2	3a	3a
Turkey	2	3b	3a
Turkmenistan	1	1	1
Tuvalu	1	1	1
Uganda	4	4b	4a
Ukraine	3	3a	4a
United Arab Emirates	3	3a	2
United Kingdom	4	4b	4b
Uruguay	3	4a	4a
USA	4	4b	4b
Uzbekistan	2	1	2
Vanuatu	1	1	1
Vatican City	2	2	1
Venezuela	3	3a	3a
Vietnam	3	3a	3a
Yemen	1	1	1
Zambia	3	4a	3b
Zimbabwe	3	4a	4a

a The 2006 World Map used a four-category system of classification; the 2011 and 2017 studies used six categories.

b These countries were not included in the 2006 and 2011 surveys.

**Supplementary Figure 3:**
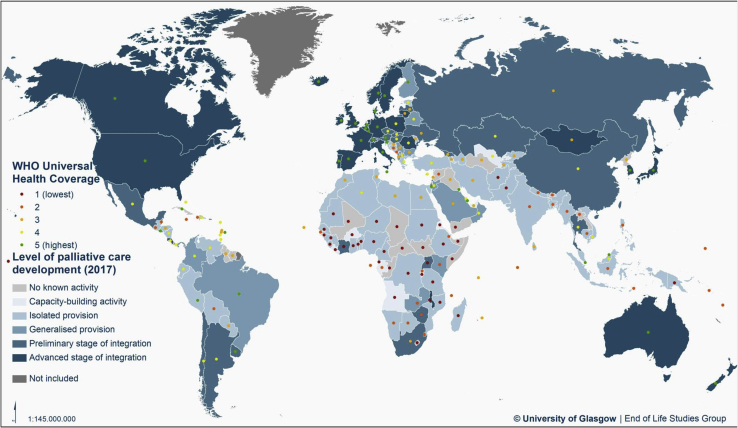
Levels of palliative care development by WHO Universal Health Coverage Index Quintile.
